# Monitoring and Failure Analysis of Corroded Bridge Cables under Fatigue Loading Using Acoustic Emission Sensors

**DOI:** 10.3390/s120403901

**Published:** 2012-03-26

**Authors:** Dongsheng Li, Jinping Ou, Chengming Lan, Hui Li

**Affiliations:** 1 School of Civil Engineering, Dalian University of Technology, No. 2, Linggong Road, Dalian 116024, China; E-Mail: oujinping@dlut.edu.cn; 2 School of Civil Engineering, Harbin Institute of Technology, 92 Xidazhi Street, Harbin 150090, China; E-Mail: lihui@hit.edu.cn; 3 School of Civil and environmental Engineering, University of Science & Technology Beijing, 30 Xueyuan Road, Beijing 10083, China; E-Mail: langchengming@gmail.com

**Keywords:** bridge cable, acoustic emission, wavelet packet, corroded, fatigue damage

## Abstract

Cables play an important role in cable-stayed systems, but are vulnerable to corrosion and fatigue damage. There is a dearth of studies on the fatigue damage evolution of corroded cable. In the present study, the acoustic emission (AE) technology is adopted to monitor the fatigue damage evolution process. First, the relationship between stress and strain is determined through a tensile test for corroded and non-corroded steel wires. Results show that the mechanical performance of corroded cables is changed considerably. The AE characteristic parameters for fatigue damage are then established. AE energy cumulative parameters can accurately describe the fatigue damage evolution of corroded cables. The failure modes in each phase as well as the type of acoustic emission source are determined based on the results of scanning electron microscopy. The waveform characteristics, damage types, and frequency distribution of the corroded cable at different damage phases are collected. Finally, the number of broken wires and breakage time of the cables are determined according to the variation in the margin index.

## 1. Introduction

Cables are the most critical bridge components and play an important role in cable-stayed systems. However, corrosion and fatigue damage easily occur, thereby reducing the security of cable-stayed bridges [1,2]. Corrosion changes the mechanical properties of a bridge cable and reduces its fatigue life. At present, several studies on bridge cable fatigue are based on non-corroded wires. A large number of studies have also focused on the effect of wire surface quality on the fatigue properties of bridge cables, as well as on analysis of their mechanical properties. However, according to the inspection results, bridge cable damage, including stress corrosion cracking, pitting, corrosion fatigue and hydrogen embrittlement, lead to the reduced service life of bridge cables [3,4]. Therefore, the development of a nondestructive method to test and evaluate the fatigue performance of cables and ensure the integrity and safety of a bridge is highly important.

Several detection methods for fatigue in bridge cables are available, including visual inspection, magnetic flux leakage testing, X-ray testing, ultrasonic testing, and cable stress detection [5–8]. Visual inspection is frequently used as a daily inspection technique, even though quantifying the damage using this method is more difficult. Magnetic flux leakage testing can identify the extent of damage and its location along the cable length, but cannot identify the location of the damage within cross-sections. X-ray examination is an efficient method of detecting the damages in cables, however, safety, cost, and portability of the necessary equipment have significantly limited its use. Ultrasonic testing can be used to detect broken wires, but stress waves transmission in wires is complex and the stress waves are significantly attenuated. Hence, ultrasonic testing is not a practical method of evaluating cable damage. The aforementioned testing methods are not ideal mainly because the wire diameter is small, and the surface roughness and region of fatigue crack are not fixed. In addition, these detection methods cannot capture the dynamic process of fatigue crack generation and expansion, their detection speed is slow, and their use entails the interruption of traffic under special circumstances.

Acoustic emission (AE) is a dynamic nondestructive detection method that has been widely used to detect the deformation, fracture and phase transition of various materials, based on the characteristics of the emitted sound waves and the external conditions that generate AE signals [9–11]. It can be used in the online monitoring of bridge cable damage and provide forecasts of early damage. The sensor layout in the AE monitoring system is very simple and it can be fixed on the surface of wires without relative movement between the wires and the detection device. Casey *et al.* [12–14] used the AE amplitude and frequency distribution to study the fatigue damage generation of wire ropes and found a one-to-one relationship between the high-amplitude signal and the broken wire. The attenuation of the broken wire AE signal amplitude is not serious after the 30-meter signal transfer. Yuyama [15] studied two continuously AE-monitored highway bridges in service for 24 days. AE was proven a very useful technique for detecting and evaluating the failures of high-strength steel tendons in prestressed concrete bridges. Drummond [16] studied the relationship between AE signal characteristics and wire breaks and found that the most effective acoustic signal discriminators are the energy and amplitude. Woodward [17] studied the wire breakage behavior near the sockets of various suspension-bridge hanger cables. The test results showed a good correlation between the recorded AE events and the wire breaks. At present, waveform analysis technology based on AE signals is rapidly developing. Antolino [18,19] applied AE technology monitoring to examine the damage evolution of corroded galvanized steel coating. The AE wavelet power parameter is suitable for the identification of damage mechanisms. Khamedi [20] studied the application of wavelet-based AE signal processing in micromechanisms to identify failure in dual phase steel.

Considerable research has been done on the AE technique for the fatigue damage monitoring and AE signals processing. In the present study, AE technology is introduced to study the fatigue damage evolution of the corroded cable of TianJin YongHe Bridge in China. The aims of this research are to identify the defects by analyzing AE signals; to understand the mechanism of crack generation, expansion, and damage generation; and to find an effective method of determining the fatigue damage evolution of corroded bridge cables.

## 2. Analysis for the Mechanical Properties of Corroded Wires

### Mechanical Properties of Corroded Wire

**Figure 1.**
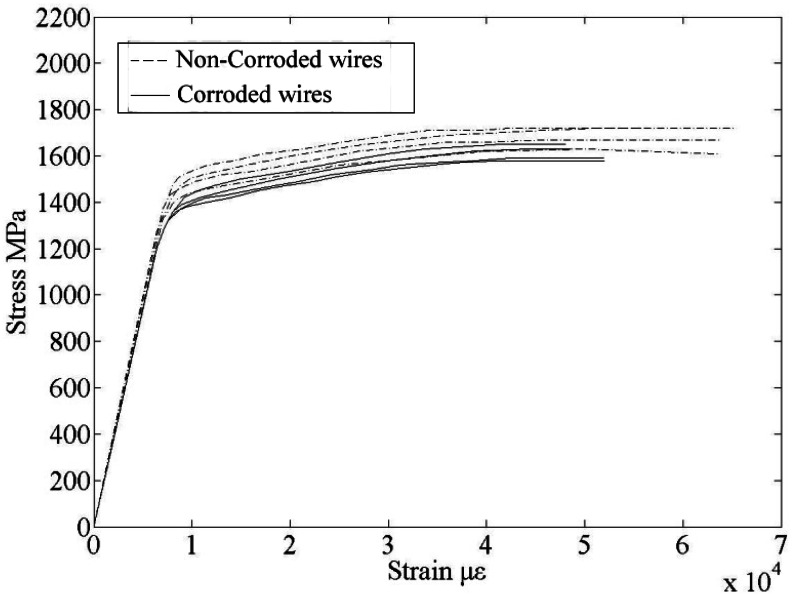
Relationship between stress and strain of wires.

The performance of a cable degrades with the passage of time, and its bearing capacity is reduced in the actual conditions. The mechanical properties of corroded steel wires from a selection of dismantled stay cables of the 18-year-old Tianjin Yonghe Bridge were examined. The length of the steel wire was 500 mm, and the test loading rate was 100 N/s. The chemical composition of the steel wires is given in Table 1. The relationship of the stress to the strain was determined through a tensile test for corroded and non-corroded steel wires. The results are shown in Figure 1.

By analyzing the experimental results, the elastic modulus of the corroded and non-corroded steel wires were shown to be the same. Corrosion had less effect on the elastic modulus; thus, this effect could be ignored. The mean nominal yield strength and nominal ultimate strength were decreased by 4%, the mean ultimate strain was reduced by 11.10%, and the percent of elongation decreased by 9.37%. Hence the elastic modulus and steel wire strength are not sensitive to corrosion, but ductility is highly sensitive. Moreover, the deformation performance of the corroded wire is very poor based on the scanning electron microscopy (SEM) images of the corroded and non-corroded steel wires fracture characteristics.

## 3. Fatigue Experiment and the AE Test for the Corroded Bridge Cable

### 3.1. Experimental Scheme

The results in Section 2 showed that corrosion reduced the ultimate strain and elongation of bridge cable, however, these two factors have great influence on steel wire fatigue performance. The steel wire mechanical properties undergo some changes due to corrosion, so characteristic fatigue damage AE signals are different with non-corroded steel wires. Currently, there is a dearth of studies on the corroded bridge cable using AE techniques. The research on corroded bridge cable fatigue damage evolution using AE signals is helpful to explain failure mechanism(s) and provide an effective method to determine its safety state. It is necessary to do fatigue testing for corroded bridge cable and offer a research base to assess the safety and predict the remaining life for the bridge cable.

A total of 69 wires were chosen from dismantled stay cables of the 18-year-old TianJin YongHe Bridge. In order to judge damage state according to AE signals, the selected steel wires should have different corrosion degree through the surface detection. In the re-manufactured new stay cable process, from outside to inside, the wires were ranged according to corrosion degree from serious to slight. This would ensure failure occurred first in the outside wires during the fatigue testing. The outside wires' fatigue crack initiation and propagation can be easily observed through visual inspection. The length of the steel wires is 1,750 mm. These steel wires were re-manufactured into new stay cables in Liuzhou OVM Co., Ltd. (China). The cable specimen was fixed on the MTS fatigue testing machine. Force was used to control the fatigue test. All specimens were tested under sinusoidal cyclic loading at a frequency of 5.0 Hz. The initial stress amplitude is 360 MPa, and the stress ratio is 0.5.

**Figure 2.**
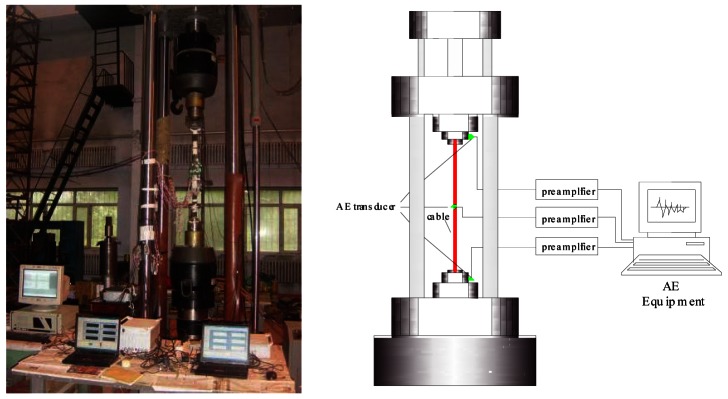
Schematic diagram of the experiment.

AE technology was adopted to monitor the damage evolution of the corroded bridge cable. The AE eight-channel AE equipment was manufactured by the Physical Acoustics Corporation. The AE parameters (hits, energy, amplitude, waveform, risetime and duration, and so on) were collected via the AEwin software. Three AE transducers were installed in the cable specimen to assess the integrality of the structure. Two R15 AE transducers were installed on the anchor as alarm transducers, filtering the signals generated from the friction between the anchor and the clamp. The AE transducers were fixed using a magnetic mechanical clamp. The third wideband AE S9208 transducer was installed in the middle of the specimen and was designed for receiving the signal generated by the cable fatigue damage of the cable. A ferrule was attached to the middle of the cable to fix the AE transducer, which was mounted on the ferrule. Prior to the fatigue experiment, a broken lead experiment was performed to test and ensure the AE transducers have the same sensitivity. The instrument settings were set at a fixed threshold level of 40 dB, a preamplifier gain of 40 dB, main amplifier gain of 20 dB, a sampling rate of 2 MHz, and a filter frequency range from 50 kHz to 1 MHz, peak definition time (PDT) of 200 μs, hit definition time (HDT) of 800 μs, hit lock time (HLT) of 1,000 μs. A schematic diagram of the experimental equipment is shown in Figure 2.

**Figure 3.**
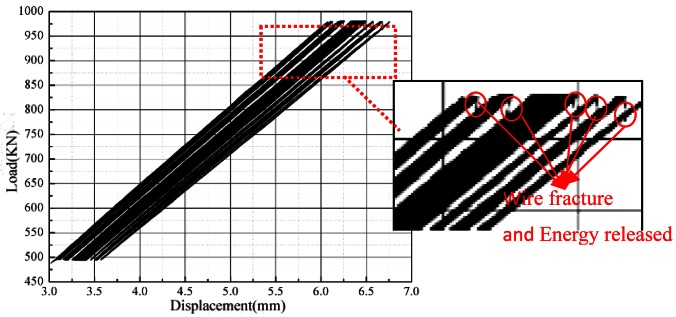
Relationship between load and displacement.

Before cable fatigue testing, the cable was tensioned in advance with a 500 kN static loading to investigate the strain difference of the different wires. The mean wire strain was 1,896.18 με and the maximum strain difference of the wire was less than the 3% of the average strain. Therefore, each wire stress was uniform based on these data. The fatigue experiment ignored the effect of nonuniform stress on the fatigue life of the wires. The load-displacement curve of the fatigue performance experiment is presented in Figure 3. Energy released from the wire fracture is clearly reflected on the load-displacement curve. After fracture, the tensile rigidity was reduced and the displacement increased because of the unchanged loading. Wire failure indicates the mutation of the displacement.

### 3.2. Analysis of the Fatigue Damage Evolution of the Entire Corroded Bridge Cable

**Figure 4.**
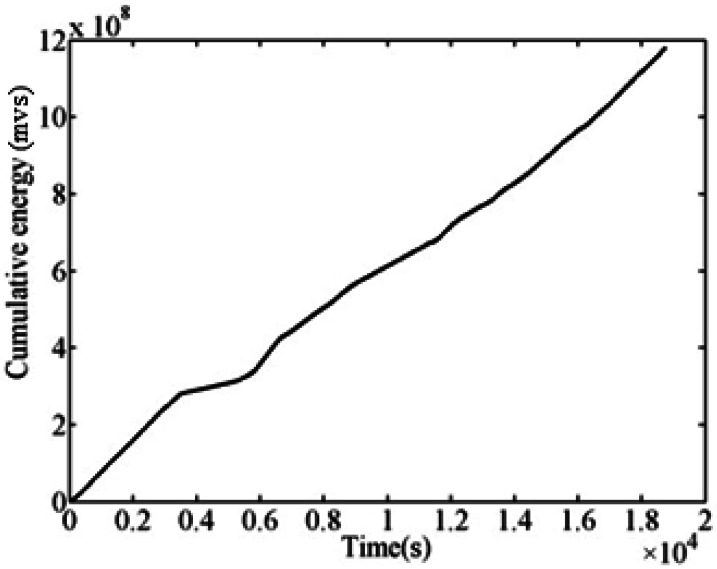
Cumulative AE energy of steel wires.

Drummond [16] applied the AE technology to study the wire fatigue damage and fracture process. He found that the energy parameters of AE can reflect the changes of in material performance because they are related to the strain energy of dislocations, fractures, and crack propagation. Therefore, the energy parameters of AE were used to describe the changes in the performance of fatigue process in the present study. The AE signal of the corroded bridge cable was abundant during the entire fatigue damage process. Figure 4 clearly shows the evolution process of the whole fatigue damage of the corroded bridge cable.

**Figure 5.**
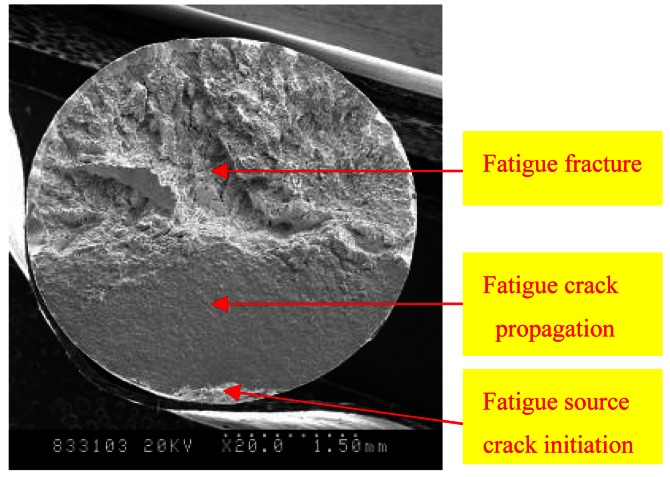
SEM images of fatigue fracture.

The fatigue fracture characteristic and fracture mechanism of steel wires were studied and analyzed using scanning electron microscopy (SEM). The SEM image of the fatigue fracture is shown in Figure 5. Fatigue fracture has three different morphological characteristics zones, namely, fatigue source crack initiation, fatigue crack propagation and failure. The source regions of fatigue cracks are the corrosion pits.

Based on the SEM images, the AE statistical characteristics parameter mean value of the different stages of the corroded bridge cable fatigue damage was integrated in Table 2.

Low-amplitude and low-energy AE signals appeared during the initial fatigue stage of the corroded bridge cable. The AE characteristic parameters rapidly increased with fatigue crack propagation, particularly at the fatigue fracture zones.

## 4. AE Typical Waveform Characteristics during the Different Stages of Fatigue Damage of the Corroded Bridge Cable

Many factors can generate AE signals during the generation of cable fatigue damage, such as metal plastic deformation, crack formation, crack propagation, fractures, and so on. The different damage mechanisms of the AE signal have different waveforms and frequency characteristics. The wavelet packet transform method was used to obtain relevant information from the AE signals to distinguish the various types of damage.

### 4.1. AE Signal Energy Feature Extraction Algorithm Using the Wavelet Packet

The 2*^j^* sub-band can be obtained when the AE signal *s* is decomposed into *j* levels using the wavelet packet. The energy of each sub-band is calculated using the square of the wavelet coefficients, as follows:

(1) $$e(j,k)=\sum_{i=1}^{N}{\left[d_{i}(j,k)\right]}^{2}$$

where *j* is the resolution level, *k* is the number of sub-bands, *N* is the signal length, and *d* is the wavelet coefficients.

The wavelet packet energy spectrum vector *E* (*j*,*s*) of signal *s* in the resolution level *j* is defined as:

(2) $$E(j,s)=[e(j,0),e(j,1),e(j,2),\ldots ,e(j,e^{j}–1)]$$

where *e* is an energy component of the sub-bands.

The energy percentage of the characteristic frequency bands as the characteristic parameters of the AE source is defined as:

(3) $$I_{k}=\frac{e(j,k)}{\sum e(j,k)}$$

where *e*(*j*,*k*) is the energy of the damage characteristics frequency bands *k* of the wavelet packet energy spectrum, and Σ *e*(*j*,*k*) is the sum of all band energies.

When the signal characteristics change, the wavelet packet energy spectrum in the same frequency band also changes. The energy of some bands is reduced and those of the other bands will increase, enabling the determination of the signal characteristics through the energy percentage difference.

### 4.2. Typical AE Waveform of the Bridge Cable Fatigue Damage

The waveform shape depends on the cracking mode, enabling the classification of cracks in different materials [21]. The wavelet packet level of the decomposition is determined by the signals themselves, and specialized instrumentation is used to acquire data.

**Figure 6.**
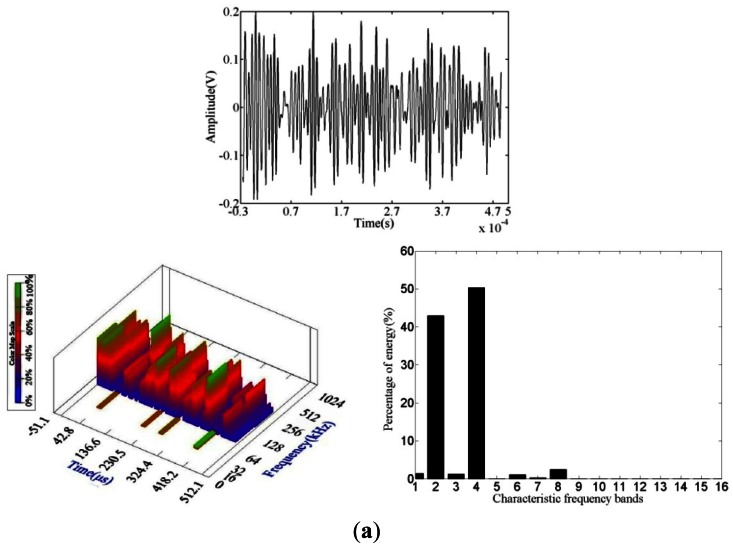

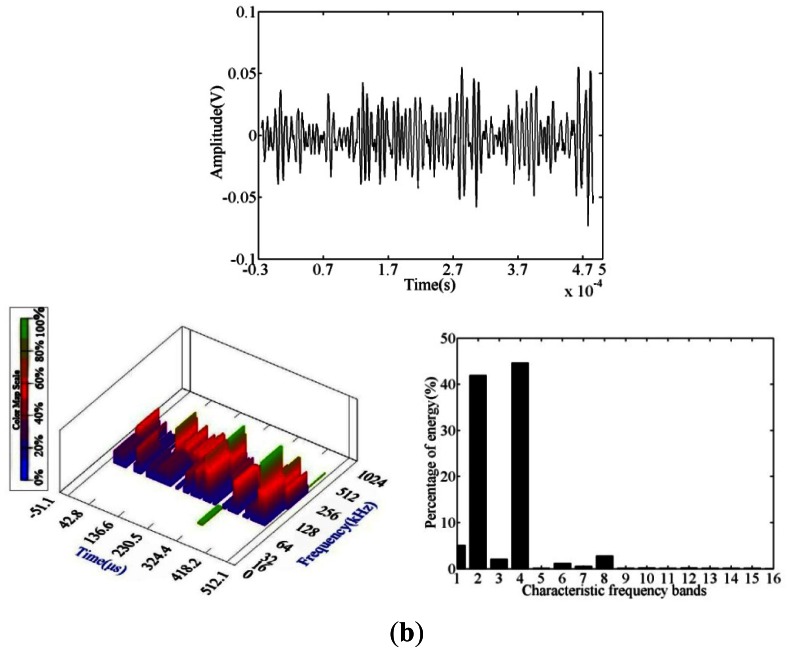
Fatigue source crack initiation AE waveform and wavelet packet analysis. (**a**) Waveform 1; (**b**) Waveform 2.

**Figure 7.**
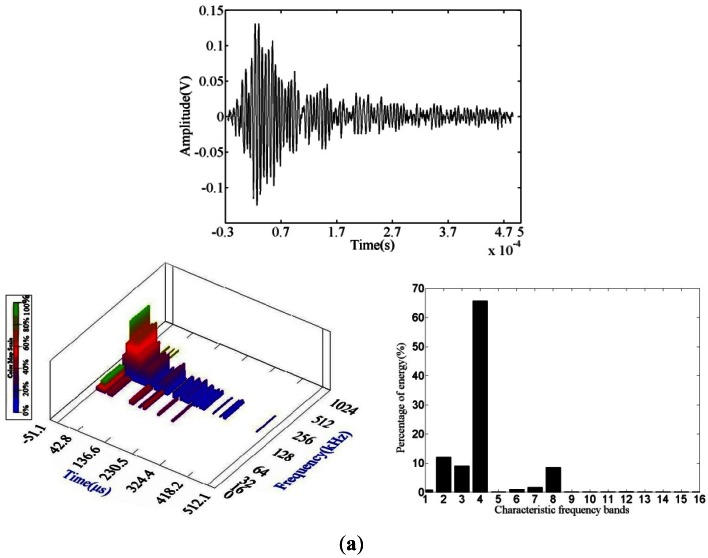

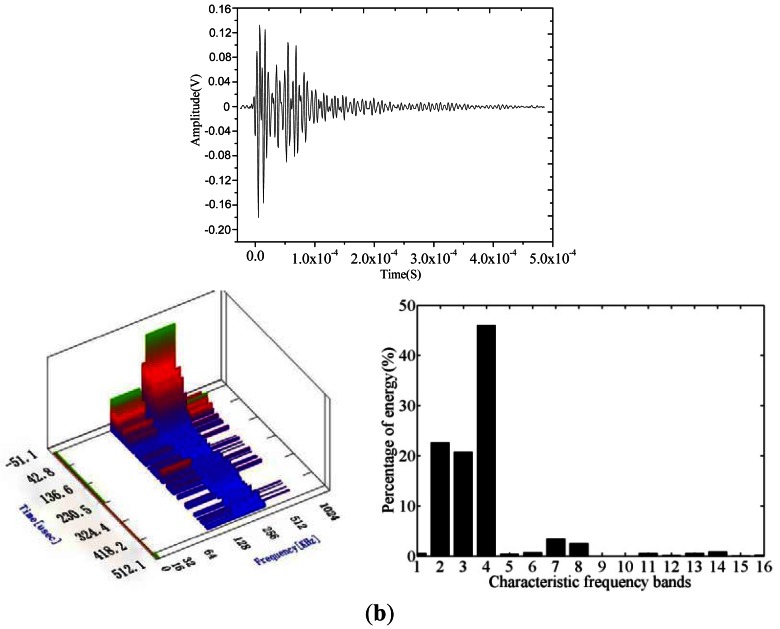
Fatigue crack propagation AE waveform and wavelet packet analysis. (**a**) Waveform 1; (**b**) Waveform 2.

**Figure 8.**
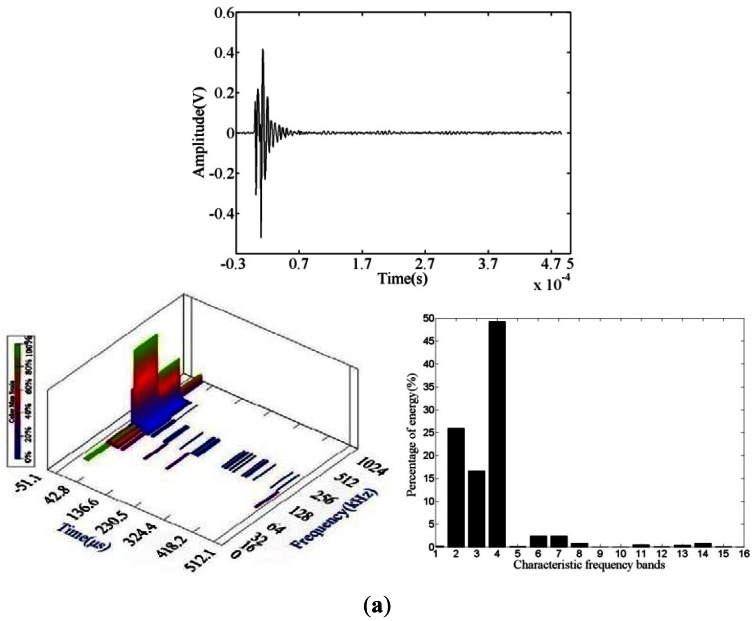

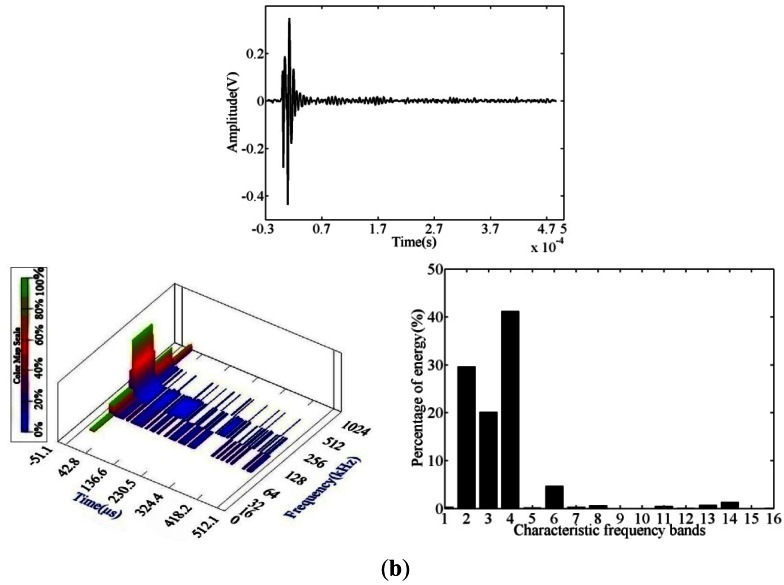
Steel wires failure AE waveform and wavelet packet analysis. (**a**) Waveform 1; (**b**) Waveform 2.

The Daubechies family of wavelets is the most popular system in wavelet transform applications, and exhibits orthogonality and compact support. It also displays rapid attenuation in the frequency domain, so as to effectively present a singular AE signal, therefore the Daubechies wavelet was used as the basis for the analysis of monitored data. In this paper, the db5 wavelet base was chosen. The AE sampling rate in the corroded cable fatigue testing is 2 MHz, and the signal Nyquist frequency is 1 MHz. Assuming that the AE signal was decomposed into six levels, and given the algorithm of the wavelet packet analysis using a binary-scale transformation, the AE signal was decomposed into 64 sub-bands, with the lowest band at 0 kHz to 15.625 kHz. Figure 5 shows that fatigue damage in the corroded cable can be classified into three stages. The fatigue damage produced at each stage and the different damages of AE signal have different damage areas. Based on the SEM images of the fatigue fracture, two typical/characteristics waveforms are selected and presented for each stage of cracking. The typical signal waveforms of the different types of damages are obtained and shown in Figures 6 to 8. The energy percentages for the first 16-order band of characteristic damage waveforms were calculated via wavelet packet analysis and are shown in Figures 6 to 8. Through AE waveform analyzing, theirs amplitude and shapes have some difference at the same damage stage, but theirs frequency and wavelet energy scale distribution are very similar. This is the reason that we adopted wavelet packet techniques analyzing AE waveform to identify damage types.

Figure 6 shows that the fatigue source waveform is a low-amplitude wide pulse with a narrow frequency scale and energy mainly concentrated in frequency bands 2 and 4, which are mostly located at 100 kHz to 200 kHz. At this stage, the AE signals are generated by the growth of surface corrosion cracks under fatigue loading, and local plastic deformation and microcracks form when the surface local stress is concentrated.

The main waveform types of fatigue crack propagation are shown in Figure 7. Through damage waveform statistics, the main damage was found to be at the point of propagation of the corroded crack. The AE signals were generated by the new area of plastic deformation damage as well as the expansion of the original corroded defects with fatigue process development. Compared to the waveform of the fatigue source, the frequency and energy scale of the damage waveform became wider, and its high frequency increased.

Under fatigue loading, the preferential slip systems of the steel wires evolved from the dominant crack through strain accumulation via crack initiation and propagation. When the steel wire entered the rapid crack propagation stage, the energy of the AE signal increased until the cable completely failed, and the AE waveform amplitude is higher than those of other stages (Figure 8). The waveform characteristic shows that this type of AE signal is a burst signal. The fracture waveform is a high-amplitude narrow pulse, with a wide energy scale, and a main frequency scale of 62.5 kHz to 875 kHz.

## 5. Analysis of the Fracture Signal of the Corroded Bridge Cable under Fatigue Loading

In machine fault diagnosis, the parameters in the time domain amplitude of the statistical analysis are used for fault detection. The diagnostic method is roughly divided into two categories: namely, the parameter of the dimension amplitude, and the parameter of the dimensionless amplitude. The parameter of the dimension amplitude is not sensitive to the change in the amplitude and frequency, whereas that of the dimensionless amplitude is independent of external factors, making it a good diagnostic parameter, thus, the parameter of the dimensionless amplitude was used to detect the damage of the AE signal in this section.

Generally speaking, the diagnostic indices have a margin index *F_MI_*, a peak index *F_PEI_*, and a pulse index *F_PUI_*. x_1_, x_2_,…, x_i_ (*i* = 1, 2, 3,…, *N*) are a series of AE signal characteristics parameters (count, energy, amplitude, and hit) in the time domain. The function for the estimated value of the parameters in the time domain amplitude is presented as follows:

(4) $$\text{Margin index}:F_{MI}=\frac{x_{\max}}{{\left(\frac{1}{N}\sum_{i=1}^{N}\sqrt{\left|x_{i}\right|}\right)}^{2}}$$

(5) $$\text{Peak index}:F_{\text{PEI}}=\frac{x_{\max}}{(\frac{1}{N}\sum_{i=1}^{N}x_{i}^{2})}$$

(6) $$\text{Pulse index}:F_{\text{PUI}}=\frac{x_{\max}}{\frac{1}{N}\sum_{i=1}^{N}\left|x_{i}\right|}$$

where the peak value x_max_ = *E*[max|x_i_|], and *N* is the signal length.

Considering that the energy parameters of AE can reflect the change in material performance, the AE energy data of the entire fatigue process were equally divided into 100 sub-intervals to simplify the calculations, and the three diagnostic indices of each sub-interval were calculated. The time of the AE fracture signal was determined by the mutation of the diagnostic indices, which shows the number of cycle loading for the fractures.

**Figure 9.**
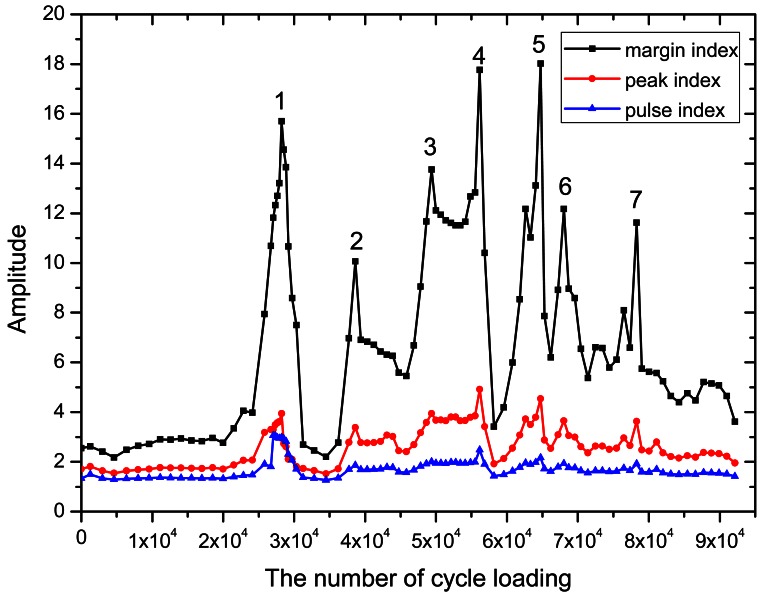
The parameters in the time domain amplitude under different load cycles. Peak value 1, 2, 3, 4, 5, 6, and 7 presented the numbers of the fracture.

Figure 9 presents the fluctuation of the margin index that can effectively show the change in the damage. The peak corresponds to the time of fracture. Actually, if the breakage of the wire occurred, it would release a huge energy (Figure 3) and the AE signal had a burst change. In the entire signal analyzing process, it must appear a peak point. Peaks value height was only shown the amount of broken wire energy. Thus, in the course of identification, the margin index is the best for damage identification, whereas the pulse and peak indices are considered the worst. However, the wire breakage ratio reached 10% when the number of cyclic loadings reached 79,005. This is because the wire suffered environmental corrosion and excessive loads for many years, causing damages/defects to the wire surfaces and leading to stress concentration on the defects. These fatigue cracks extended from the surface, resulting in significantly reduced fatigue life.

## 6. Conclusions

In the present study, corroded bridge cables from the TianJin YongHe Bridge were subjected to fatigue experiments using the AE technique. The main conclusions are as follows:

1. The steel wires elastic modulus was not sensitive to corrosion, but nominal yield strength, nominal ultimate strength and elongation rate were highly sensitive. The corroded steel wire fatigue performance had some difference with respect to uncorroded steel wire.
2. The AE characteristic parameters were determined through corroded bridge cable fatigue experiments. The test results show that the fatigue damage evolution process of the corroded bridge cable can be expressed according to the curves of the cumulative AE energy.
3. The types of damage at the different stages were identified based on SEM images of the fatigue fracture. The AE statistical parameters and waveform characteristics were combined to analyze the the characteristics of the the AE source. The different damage mechanisms of the AE signals have different waveform characteristics and frequency scales.
4. The AE signals of broke wire can be evaluated by calculating the fault detection indices of the AE parameter signals. The margin index is more sensitive compared to other indices. The load cycles can also be reported when the wire fails. The results show that the life of the corroded cable is shorter than the standard life of uncorroded cables.

The results in this paper are based on the signals received by a channel and were not obtained from a combination of the best of results of other AE channel signals. An effective method of combining multi-channel information is to highlight the principal component signal and increase the accuracy of the analytical results. This approach should be the main direction of future studies.

## Figures and Tables

**Table 1.** Chemical compositions (wt.%) of steel wires.

**Compositions**	**C**	**Si**	**Mn**	**P**	**S**	**Cu**
wt.%	0.75∼0.85	0.12∼0.32	0.60∼0.90	≤0.025	≤0.025	≤0.20

**Table 2.** The AE signal mean values characteristic parameters range at different fatigue damage stages.

**AE parameters**	**Fatigue source crack initiation**	**Fatigue crack propagation**	**Fatigue fracture**
Amplitude (dB)	55	75	90
Energy (mv.s)	125	245	482
Count	157	263	507
Rise time (μs)	373	605	1,120
Duration (μs)	3,221	3,576	661
